# Differences in Muscle Protein Synthesis and Anabolic Signaling in the Postabsorptive State and in Response to Food in 65–80 Year Old Men and Women

**DOI:** 10.1371/journal.pone.0001875

**Published:** 2008-03-26

**Authors:** Gordon I. Smith, Philip Atherton, Dennis T. Villareal, Tiffany N. Frimel, Debbie Rankin, Michael J. Rennie, Bettina Mittendorfer

**Affiliations:** 1 School of Medicine, Washington University, St. Louis, Missouri, United States of America; 2 School of Graduate Entry Medicine and Health, University of Nottingham, Derby, United Kingdom; Universidad Europea de Madrid, Spain

## Abstract

Women have less muscle than men but lose it more slowly during aging. To discover potential underlying mechanism(s) for this we evaluated the muscle protein synthesis process in postabsorptive conditions and during feeding in twenty-nine 65–80 year old men (n = 13) and women (n = 16). We discovered that the basal concentration of phosphorylated eEF2^Thr56^ was ∼40% less (*P*<0.05) and the basal rate of MPS was ∼30% greater (*P* = 0.02) in women than in men; the basal concentrations of muscle phosphorylated Akt^Thr308^, p70s6k^Thr389^, eIF4E^Ser209^, and eIF4E-BP1^Thr37/46^ were not different between the sexes. Feeding increased (*P*<0.05) Akt^Thr308^ and p70s6k^Thr389^ phosphorylation to the same extent in men and women but increased (*P*<0.05) the phosphorylation of eIF4E^Ser209^ and eIF4E-BP1^Thr37/46^ in men only. Accordingly, feeding increased MPS in men (*P*<0.01) but not in women. The postabsorptive muscle mRNA concentrations for myoD and myostatin were not different between sexes; feeding doubled myoD mRNA (*P*<0.05) and halved that of myostatin (*P*<0.05) in both sexes. Thus, there is sexual dimorphism in MPS and its control in older adults; a greater basal rate of MPS, operating over most of the day may partially explain the slower loss of muscle in older women.

## Introduction

Adequate maintenance of muscle mass throughout life is important to preserve locomotor functions and diminish the risk of falling. Furthermore, muscle is the predominant site of body glucose uptake [1]–[3] and contributes ∼25% to basal energy expenditure, and even more during physical activity [4]. Muscle, therefore, not only serves mechanical functions but also contributes to metabolic and energy homeostasis.

It is common knowledge that healthy adult women have less lean body mass (mostly muscle) and more fat than men [5], [6]. However, the age-associated decrease in lean body mass and muscle mass is slower in women than in men [5], [7]–[9]. Unfortunately, little is known about the mechanisms that lead to sexual dimorphism in body composition. It is thought that most of it is due to differences in the sex-hormone milieu in men and women. It has been repeatedly demonstrated *in vitro* [10], [11], *in vivo* in animal models [10], [12]–[14], and in human subjects [15], [16] that testosterone stimulates skeletal muscle protein synthesis (MPS) and increases muscle mass. There is also evidence that ovarian hormones inhibit MPS [17] and muscle growth [18], [19] in rats. Nevertheless, several investigators who have measured the rates of MPS in men and women have found no differences [20]–[22].

The fact that no sex differences in MPS have been reported in the literature might be because these studies were conducted in young and middle-age adults with a constant muscle mass during postabsorptive conditions, when sex differences may be small or non-existent. We reasoned that sex differences in muscle protein turnover would become apparent at life stages when muscle mass is changing (e.g., during growth in adolescence or wasting during aging) and/or during acute anabolic or catabolic challenges (e.g., feeding or injury). Accordingly, we hypothesized that such differences might be revealed by a comparison of rates of MPS in older men and women during basal, postabsorptive conditions and feeding. We, therefore, measured the fractional rate of MPS during basal, postabsorptive conditions and during feeding by using stable-isotope labeled amino acid tracer techniques in 65–80 year old men and women; we also measured the concentrations of total muscle RNA and protein to gain insight into the protein synthetic capacity and translational efficiency of the muscle [23], [24]. Furthermore, we measured the activation (as phosphorylation) of elements of intracellular signaling pathways involved in the regulation of MPS (Akt; ribosomal protein S6 protein kinase [p70s6k]; eukaryotic initiation factor 4E [eIF-4E]; eIF4E binding protein 1 [eIF4E-BP1]; and eukaryotic elongation factor 2 [eEF2]) [25], [26] and the mRNA expression of proteins involved in the regulation of muscle mass (i.e., the muscle growth inhibitor myostatin [27], [28] and the muscle growth factor myoD [29]). We also measured plasma C-reactive protein (CRP) concentration as an index of inflammation because the concentrations of CRP and pro-inflammatory cytokines in blood have been found to be negatively associated with rates of MPS [30] and may contribute to skeletal muscle atrophy and reduced functional capacity, especially during aging [31]–[33].

## Methods

### Subjects

We studied 13 men and 16 women, aged 65 to 80 y; men and women were matched for age and body mass index (Table 1). All subjects were considered to be in good health after completing a comprehensive medical evaluation. None of the subjects engaged in regular exercise, reported excessive alcohol intake, smoked, or received hormone replacement therapy. Ten women and six men were treated for hypertension, and four women and four men were treated for hypercholesteremia; the drug regimen had been initiated several years before subjects entered the study and had been stable for several months before beginning the study. Written, informed consent was obtained from all subjects before their participation in the study, which was approved by the Human Studies Committee and the General Clinical Research Center (GCRC) Advisory Committee at Washington University School of Medicine in St. Louis, MO.

**Table 1:** Subject characteristics

	Men	Women	P-value
Age (years)	71±2	69±1	0.16
Body mass index (kg·m^−2^)	36±1	38±2	0.34
Body mass (kg)^a^	108±3	98±4	0.09
Fat free mass (kg)^a^	67±2	51±2	<0.001
Fat free mass (% body weight)	62±1	52±1	<0.001
Appendicular muscle mass (kg)^a^	29±1	22±1	<0.001
Appendicular muscle mass (% FFM)	43±1	43±1	0.40
Thigh muscle volume (cm^3^)^b^	2477±113	1963±93	0.002

Values are means±SEM.

a Measured by DEXA as described in the Experimental protocol section.

b Measured by MRI as described in the Experimental protocol section.

### Experimental protocol

Approximately two weeks before the protein metabolism study, subjects' total body fat-free mass (FFM) and appendicular muscle mass [31] were measured by using dual-energy X-ray absorptiometry (Delphi-W densitometer, Hologic, Waltham, MA). Magnetic resonance imaging (MRI) was used to quantify thigh muscle volume; images were acquired with a 1.5-T superconducting magnet (Siemens, Iselin, NJ) and a T1-weighted pulse sequence. Eight 8-mm-thick axial images, starting 10 cm proximal to the distal edge of the femur, with a 7-mm intersection gap, were acquired and muscle volume in each of the images was determined with the NIH Image Analysis Software (Analyze Direct software (version 7.0; Mayo Clinic, Rochester, MN), which utilizes pixel brightness to distinguish muscle from other tissues. Thigh muscle volume in the region of interest was calculated as the sum of the individual muscle volumes and the sum of the muscle volumes in the intersection gap, which were assumed to be the same as in the preceding image.

Three days before the protein metabolism study, subjects were instructed to adhere to their usual diet and to refrain from vigorous exercise until completion of the study. The evening before the study, subjects were admitted to the GCRC. At 2000 h, they consumed a standard meal which provided 50.2 kJ per kg body weight; 15% of the meal energy was provided as protein, 55% as carbohydrates and 30% as fat. Subjects then rested in bed and fasted (except for water) until completion of the study the next day. At ∼0600 h on the following morning, a cannula was inserted into an antecubital vein for the infusion of a stable isotope labeled leucine tracer; another cannula was inserted into a vein of the contralateral hand for blood sampling. At ∼0800 h, a blood sample and a muscle biopsy from the quadriceps femoris were obtained to determine the background leucine enrichment in plasma, the concentrations of testosterone, progesterone, 17ß-estradiol, sex hormone binding globulin (SHBG), and CRP in plasma, the background leucine enrichment in muscle tissue fluid and muscle protein, and the concentration of total RNA and protein in muscle. Muscle tissue (∼50–100 mg) was obtained under local anesthesia (lidocaine, 2%) by using Tilley-Henkel forceps [34]. Immediately afterwards, a primed, constant infusion of [5,5,5-^2^H_3_] L-leucine (98 Atoms % purchased from Cambridge Isotope Laboratories Inc, Andover, MA; priming dose: 4.8 µmol kg body wt^−1^, infusion rate: 0.08 µmol kg body wt^−1^·min^−1^) was started and maintained until completion of the study ∼6 h later. At 210 min after the start of the leucine tracer infusion, a second muscle biopsy was obtained to determine the basal rate of MPS (as incorporation of [5,5,5-^2^H_3_]leucine into muscle protein; see *Calculations*) and the basal concentrations of phosphorylated elements of intramuscular signal transduction proteins (Akt; p70s6k; eIF-4E; eIF4E-BP1; and eEF2) involved in the regulation of MPS. Immediately after the second biopsy, a liquid meal (Ensure®, Abbott Laboratories, Abbott Park, IL, USA, containing 15% of energy as protein, 55% as carbohydrate and 30% as fat) was given intermittently in small boluses every 10 minutes for 150 min so that every subject received a priming dose of 23 mg protein·kg FFM^−1^ and 70 mg protein·kg FFM^−1^·h^−1^ during the 2.5 h feeding period. At the onset of feeding, the infusion rate of labeled leucine was increased to 0.12 µmol kg body wt^−1^·min^−1^ to adjust for the increased plasma leucine availability. A third muscle biopsy was obtained at 360 min (i.e., 150 min after the first food aliquot) to determine both MPS and the intracellular signaling responses to feeding. The second and third biopsies were obtained from the leg contralateral to that biopsied initially through the same incision, but with the forceps directed in proximal and distal direction so that the two biopsies were collected ∼5–10 cm apart. Blood samples were obtained every 30 min during the entire study period to determine plasma leucine enrichment and concentration, and concentrations of glucose, and insulin. The tracer infusions were stopped and cannulae were removed after the last (third) biopsy and the final blood draw were completed.

### Sample collection and storage

Approximately 4 ml of blood was collected on each occasion. One milliliter was collected in pre-chilled tubes containing heparin, plasma separated immediately by centrifugation and glucose concentration measured immediately. The remaining blood (∼3 ml) was collected in pre-chilled tubes containing EDTA, plasma was separated by centrifugation within 30 min of collection and then stored at −80°C until final analysis. Muscle samples were rinsed in ice-cold saline immediately after collection, cleared of visible fat and connective tissue, frozen in liquid nitrogen and stored at −80°C until final analysis.

### Sample processing and analyses

Plasma glucose concentration was determined on an automated glucose analyzer (Yellow Spring Instruments, Yellow Springs, OH). Plasma insulin concentration was determined by radioimmunoassay (Linco Research, St. Louis, MO). ELISA was used to determine plasma concentrations of testosterone, progesterone, 17ß-estradiol, SHBG (all Immuno-Biological Laboratories, IBL-America, Minneapolis, MN), and CRP (ALPCO Diagnostics, Salem, NH).

To determine plasma leucine concentration and labeling of plasma leucine and α-ketoisocaproate (KIC), a known amount of norleucine was added to the plasma, proteins were precipitated, and the supernatant, containing free amino acids and their keto-analogues, was collected to prepare the *t*-butyldimethylsilyl (*t*-BDMS) and trimethylsilyl derivative of leucine and KIC, respectively, to determine their tracer-to-tracee ratios (TTR) by gas-chromatography/mass-spectrometry (GC-MS; MSD 5973 System, Hewlett-Packard) [35].

To determine leucine labeling of muscle proteins and tissue fluid, samples (∼20 mg) were homogenized in 1 ml trichloroacetic acid solution (3% w/v), proteins precipitated by centrifugation, and the supernatant, containing free amino acids, collected. The pellet containing muscle proteins was washed and then hydrolyzed in 6 N HCl at 110 °C for 24 h. Amino acids in the protein hydrolysate and supernatant samples were purified on cation-exchange columns (Dowex 50W-X8-200, Bio-Rad Laboratories, Richmond, CA), and the t-BDMS derivative of leucine prepared to determine its TTR by GC-MS (MSD 5973 System, Hewlett-Packard) analysis [35]. The extent of leucine labeling in plasma, muscle tissue fluid, and muscle protein were calculated based on the simultaneously measured TTR of standards of known isotope labeling.

Western analysis was used to measure the phosphorylation of Akt, p70s6k, eIF-4E, eIF4E-BP1, and eEF2. Briefly, frozen muscle tissue (∼20 mg) was rapidly homogenized with scissors in ice-cold buffer (50 mM Tris-HCL pH 7.5, 1 mM EDTA, 1 mM EGTA, 10 mM glycerophosphate, 50 mM NaF, 0.1% Triton-X, 0.1% 2-mercaptoethanol, 1 complete protease inhibitor tablet [Roche Diagnostics Ltd, Burgess Hill, UK]) at 10 µl·mg^−1^ tissue. Proteins were extracted by shaking for 15 min at 4°C and samples were then centrifuged at 13000×*g* for 10 min at 4°C and the supernatant, containing the proteins was collected. The protein concentration in the supernatant was determined by the Bradford method with a commercial reagent (B6916, Sigma-Aldrich, St. Louis, MO) and adjusted to 3 mg ml^−1^ in 3×Laemmli buffer. Fifty micrograms of protein from each sample were loaded onto 12% XT-Bis Tris gels, separated by SDS PAGE, and transferred on ice at 100 V for 45 min to methanol pre-wetted 0.2 µm PVDF membranes. Blots were then incubated sequentially with 5% (w/v) non-fat milk for 1 h, primary antibodies overnight at 4°C, and then secondary antibody (1:2000 anti-rabbit; New England Biolabs, Ipswich, MA) for 1 h. The following primary antibodies were used at a concentration of 1∶1000: Akt^Thr308^, p70s6k^Thr389^; 4E-BP1^Thr37/46^, eEF2^Thr56^, and GADPH (loading control), purchased from New England Biolabs, and eIF4E^Ser209^ purchased from Santa Cruz Biotechnology Inc. (Santa Cruz, CA). Membranes were developed using Immunstar (Bio-Rad Laboratories, Richmond, CA) and the protein bands were visualized and quantified by densitometry on a Chemidoc XRS (Bio-Rad Laboratories, Inc. Hercules, CA) ensuring no pixel saturation. Data were expressed in relation to GADPH.

The expression of genes involved in the regulation of muscle mass was evaluated by real-time, reverse transcription polymerase chain reaction (RT-PCR). Frozen tissue samples (5–10 mg) were homogenized in TRIZOL® using a Polytron for 15 s on ice. Total RNA was extracted according to the instructions provided by the manufacturer (Sigma-Aldrich, St. Louis, MO) and quantified by spectrophotometry at 260 nm. An aliquot (0.5 µg) was loaded onto a 1% agarose gel to check RNA quality and loading by visualization of 28s and 18s rRNA. Reverse transcription was performed using the iScript synthesis kit (Bio-Rad Laboratories, Richmond, CA) with 1 µg of total RNA in a reaction volume of 20 µl (4 µl iScript reaction mix, 1 µl iScript reverse transcriptase, 1 µl RNA template, 14 µl RNase-free water). The final RT products were adjusted to 100 µl each using RNase free water. The following primers were used for myostatin and myoD (all 5′ to 3′). Myostatin forward: CTA CAA CGG AAA CAA TCA TTA CCA, reverse: GTT TCA GAG ATC GGA TTC CAG TAT; MyoD forward: CCG CCT GAG CAA AGT AAA TG, reverse: GCC CTC GAT ATA GCG GAT G. Sybr Green® PCR analyses were carried out on the iQ5 Real-Time PCR Detection System (Bio-Rad Laboratories, Richmond, CA) using the following cycle conditions: 3 min at 95°C, followed by 40 cycles of 1 min at 60°C, and 15 s at 95°C. For each gene, real time RT-PCR was conducted in duplicate in 25 µl reaction volumes containing 12.5 µl qPCR SuperMix (Bio-Rad Laboratories, Richmond, CA), 0.75 µl of each primer (10 pmol µl^−1^), 9 µl RNAse-free water and 2 µl of 1∶5 diluted cDNA. PCR products were checked for amplicon specificity by both melting curve and agarose gel electrophoresis. Results were analyzed using the 2-ΔΔCt method with 28s as internal control [36]. Due to lack of sufficient muscle tissue, these analyses were carried out in only 7 of the 13 men and 7 of the 16 women.

To determine the total RNA to protein ratio in muscle, an index of the capacity for protein synthesis, an aliquot of the total RNA preparation prepared for RT-PCR was sequentially extracted (RNA>DNA>protein) according to the manufacturers (Sigma-Aldrich, St. Louis, MO) protocol. Total RNA was quantified after complete removal of the upper phase following phase separation; protein concentration was quantified after removal of the DNA interphase and precipitation of proteins with acetone from the bottom layer, which were then washed and resuspended in 1% SDS. Total RNA was quantified (in µg per g wt weight) spectrophotometrically at 260 nm and protein was quantified (in mg per g wt weight) at 595 nm using Bradford reagents at a 1∶10 dilution to reduce SDS interference. Due to lack of sufficient muscle tissue, these analyses were carried out in only 9 of the 13 men and 7 of the 16 women.

### Calculations

Leucine rate of appearance (Ra) in plasma was calculated by dividing the rate of [5,5,5–^2^H_3_]leucine infusion by the steady state plasma KIC TTR during basal, postabsorptive conditions and feeding. Leucine Ra during basal conditions is an index of the rate of whole-body proteolysis; during feeding, leucine Ra represents the sum of the rate of leucine release into plasma from proteolysis plus the rate of transfer of absorbed leucine from the meal into the systemic circulation.

The fractional synthesis rate (FSR) of mixed muscle protein was calculated from the rate of incorporation of [5,5,5-^2^H_3_]leucine into muscle protein, using a standard precursor-product model as follows: FSR = ΔE_p_/E_ic_×1/*t*×100; where ΔE_p_ is the change between two consecutive biopsies in extent of labeling (TTR) of protein-bound leucine. E_ic_ is the mean labeling over time of the precursor for protein synthesis and *t* is the time between biopsies. The free leucine labeling in muscle tissue fluid was chosen to represent the immediate precursor for MPS (i.e., aminoacyl-*t*-RNA) [37]. Values for FSR are expressed as %·h^−1^. The absolute rate of muscle protein synthesis (g protein per hour) was calculated by multiplying the FSR by the total appendicular muscle protein mass, which was assumed to be 20% of total appendicular muscle mass. We have recently found that differences in the rates of muscle protein synthesis in different muscles are negligible[38]; thus, it is reasonable to extrapolate our data obtained in the vastus lateralis to all skeletal muscles in the body.

The translation efficiency (mg protein produced per µg RNA per hour) was calculated by dividing the product of the muscle protein FSR (in %·h^−1^) and the muscle protein concentration (in mg per g wet tissue) by the muscle total RNA concentration (in µg per g wet tissue) [23], [24].

### Statistical analysis

All data sets were tested for normality. Differences between men and women in subject characteristics and single time-point measurements (e.g., plasma sex hormone and CRP concentrations) were evaluated by using Student's t-test for normally distributed data and the Mann-Whitney U test for data which were not normally distributed (i.e., plasma SHBG, testosterone, progesterone, 17ß-estradiol and CRP concentrations). Analysis of variance (ANOVA) was used to evaluate possible differences between men and women in plasma glucose, insulin, and leucine concentrations, muscle protein FSR, muscle intracellular signaling elements, and muscle mRNA expression during postabsorptive and fed conditions. If necessary, data were log transformed to achieve normally distributed data sets before analysis. A *P* value of ≤0.05 was considered statistically significant. Data in the text are presented as mean±SEM or median with 25^th^ and 75^th^ percentiles in brackets for skewed data sets; data in tables and figures are presented as indicated in the legends.

## Results

### Subjects' age and body-composition

Men and women were matched for age and BMI (Table 1). Total body FFM, total muscle mass and leg muscle volume were ∼25% less in women than in men; however, the relative contribution of muscle mass to total body FFM was not different in men and women (Table 1).

### Plasma sex hormone and CRP concentrations

Plasma SHBG concentration was not different between men and women (Table 2). Plasma testosterone concentration was 10 times greater (*P*<0.001) in men than in women whereas plasma progesterone and 17ß-estradiol concentrations were not different between the sexes (Table 2). Plasma CRP concentration was not different between men and women (Table 2).

**Table 2:** Plasma sex hormone and CRP concentrations.

	Men	Women
SHBG (nmol l^−1^)	23.1 (20.1, 26.0)	25.9 (19.2, 44.8)
Testosterone (nmol l^−1^)	12.2 (8.8, 18.1)	1.1 (0.9, 1.7)*
Progesterone (ng ml^−1^)	0.12 (0.05, 0.31)	0.04 (0.02, 0.12)
17ß-Estradiol (pg ml^−1^)	11.3 (10.8, 30.9)	10.9 (10.0, 11.9)
CRP (mg l^−1^)	3.08 (2.43, 4.23)	2.80 (0.98, 3.60)

Values are median with quartiles in parentheses.

* Value significantly different from corresponding value in men (*P*<0.001).

### Plasma glucose, insulin, and leucine concentrations

Basal plasma glucose and insulin concentrations were not different between men and women (Table 3). Feeding increased plasma glucose concentration by ∼25% (*P*<0.001) and plasma insulin concentration by ∼200% (*P*<0.001) with no differences between the sexes (Table 3). Basal plasma leucine concentration was ∼15% less in women than in men (Table 3), and feeding increased plasma leucine concentration by ∼15% (*P*<0.001) in both sexes (Table 3).

**Table 3:** Plasma glucose, insulin, and leucine concentrations.

	Men		Women	
	Fasted	Fed	Fasted	Fed
Glucose (mmol l^−1^)	5.2±0.1	6.5±0.2†	5.4±0.2	6.9±0.1†
Insulin (µU ml^−1^)	15.5±2.2	47.8±7.3†	14.4±3.1	41.5±5.6†
Leucine (µmol l^−1^)	133±4	144±4†	111±6*	126±6* †

Values are mean±SEM.

* Value significantly different from corresponding value in men (*P* = 0.011);

† value significantly different from corresponding value during basal, postabsorptive (fasted) conditions (*P*<0.001).

### Whole-body leucine Ra

Rates of leucine Ra during basal, postabsorptive conditions (an index of whole-body protein breakdown) and total leucine Ra during feeding were not different in men (2.24±0.08 and 2.56±0.08 µmol kg^−1^ FFM·min^−1^, respectively) and women (2.20±0.09 and 2.62±0.09 µmol kg^−1^ FFM·min^−1^, respectively).

### Muscle protein synthesis

The capacity for MPS (i.e., total RNA-to-protein ratio in muscle) tended to be greater (by ∼20%; *P* = 0.18) in women than in men (5.8±0.6 vs. 4.7±0.5 µg RNA·mg protein^−1^, respectively).

**Figure 1:**
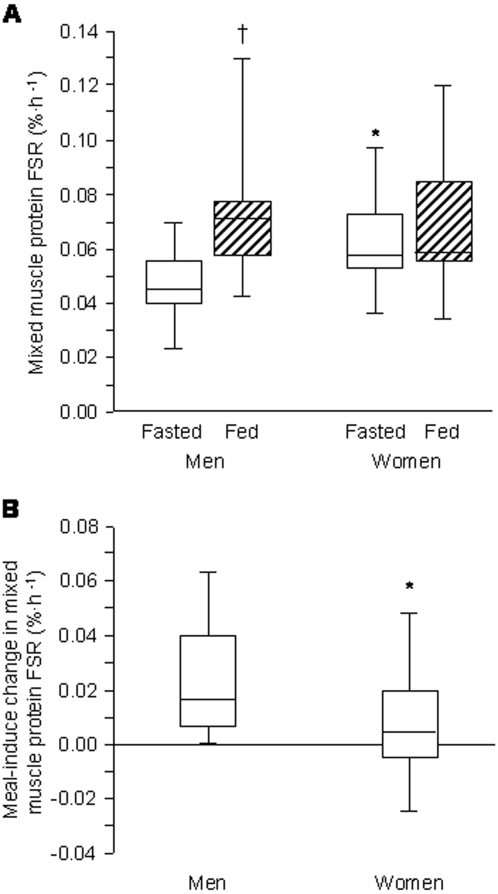
Muscle protein synthesis rate in men and women. Panels A and B show the mixed skeletal muscle protein fractional synthesis rate (FSR) during basal, post-absorptive conditions (fasted) and liquid mixed meal consumption (fed) in men and women (A) and the meal-induced change in the FSR in men and women (B). Graphs show the median (central horizontal line), 25^th^ and 75^th^ percentiles (box), and minimum and maximum values (vertical lines). * Value significantly different from corresponding value in men. ^†^ Value significantly different from corresponding value during basal, postabsorptive (fasted) conditions.

Mixed muscle protein FSR during basal, postabsorptive conditions was ∼30% greater (*P* = 0.02) in women than in men (Figure 1). Feeding had no effect on the FSR in women but increased (*P*<0.01) it in men to values similar to those in women (Figure 1). The absolute rate of muscle protein synthesis, adjusted for differences in total muscle mass between the sexes, was 116 [104, 143] mg of muscle protein per hour per kg of appendicular skeletal muscle mass in women and 90 [78, 115] mg of muscle protein per hour per kg of appendicular skeletal muscle mass in men (P = 0.02); it increased by 35 [13, 79] mg per hour per kg of appendicular skeletal muscle mass in response to the meal in men (*P*<0.01), but did not change significantly from basal values (by 11 [−10, 41] mg of muscle protein per hour per kg of appendicular skeletal muscle mass) in women.

The rate of MPS in relation to muscle RNA concentration, a measure of the translational efficiency in muscle, was not different between men and women during basal, postabsorptive conditions (0.012±0.003 vs. 0.013±0.002 mg protein·µg RNA^−1^·h^−1^) and increased with feeding in men (to 0.019±0.006 mg protein·µg RNA^−1^·h^−1^; *P* = 0.058 vs basal) but not in women (to 0.015±0.001 mg protein·µg RNA^−1^·h^−1^).

### Phosphorylation of signaling transduction proteins in muscle

**Figure 2:**
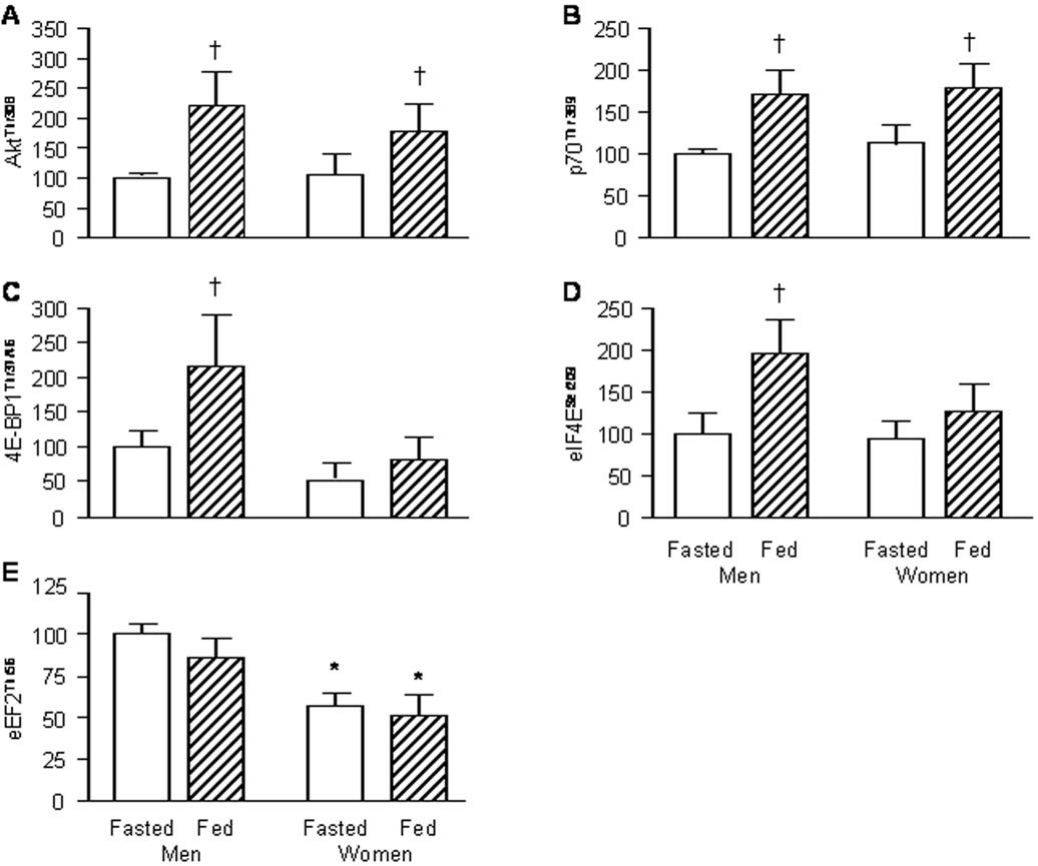
Phosphorylation of anabolic signaling transduction molecules in muscle of men and women. Panels A–E show concentrations of phosphorylated Akt^Thr308^ (A), p70s6k^Thr389^ (B), eIF4E-BP1^Thr37/46^ (C), eIF4E^Ser209^ (D), and eEF2^Thr56^ (E) during basal, postabsorptive conditions (fasted) and liquid mixed meal consumption (fed) in men and women. Values are means±SEM. * Value significantly different from corresponding value in men; ^†^ Value significantly different from corresponding value during basal, postabsorptive (fasted) conditions.

In the postabsorptive state, the extent of phosphorylation of Akt^Thr308^, p70s6k^Thr389^, eIF4E^Ser209^ and eIF4E-BP1^Thr37/46^ in muscle was not different in men and women (Figure 2). The feeding-induced increases in the phosphorylation of Akt^Thr308^, p70s6k^Thr389^ (both *P*<0.01) were also not different in the two sexes (Figure 2). In contrast, feeding increased (P<0.01) the phosphorylation of eIF4E^Ser209^ and eIF4E-BP1^Thr37/46^ in men but had no effect on their phosphorylation in women (Figure 2). Phosphorylated eEF2^Thr56^ in muscle was ∼40% less (*P*<0.05) in women than in men both during postabsorptive conditions and feeding (Figure 2); there was a tendency for a decrease in the phosphorylation of eEF2^Thr56^ in both sexes with feeding (Figure 2) but this difference did not reach statistical significance (*P* = 0.103).

### mRNA expression of proteins involved in the regulation of muscle mass

**Figure 3:**
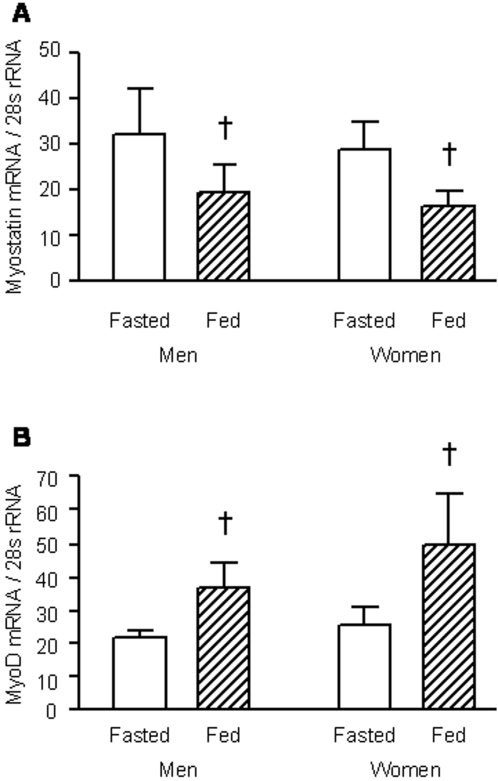
Muscle mRNA expression of proteins involved in the regulation of muscle mass in men and women. Panels A and B show myostatin (A) and myoD (B) gene expression during basal, postabsorptive conditions (fasted) and liquid mixed meal consumption (fed) in men (n = 7) and women (n = 7). Values are means±SEM. ^†^ Value significantly different from corresponding value during basal, postabsorptive (fasted) conditions; *P*<0.05.

The mRNA concentrations of myostatin and myoD during postabsorptive conditions were not different in men and women (Figure 3). Feeding decreased the concentration of myostatin mRNA (*P*<0.05) and increased the concentration of myoD mRNA (*P*<0.05) to the same extent in the two sexes (Figure 3).

## Discussion

In this study we uncovered marked sexual dimorphism between older men and women in a variety of aspects of muscle protein metabolism. The differences we observed between older men and women, namely a ∼30% greater basal rate of mixed MPS in women than in men and resistance of MPS to feeding a liquid mixed meal (providing a total of ∼10 g of protein) in women, are consistent with a recent study in which comprehensive oligonucleotide microarrays were used to discover potential differences between men and women in the expression of genes involved in the regulation of muscle mass [39]; in this study it was found that women had a two-fold greater expression of two genes that encode proteins with inhibitory properties on growth factor pathways in muscle. On the other hand, the results from our study are in clear contrast to the results from previous workers who searched for potential sex differences in human muscle protein metabolism and found none [20]–[22]. However, those earlier studies were conducted in young adults (average age: 23–27 y) and we hypothesized that sex differences in human muscle protein turnover would only become apparent at life stages when muscle mass was changing (e.g., during adolescent growth or wasting during aging) and/or possibly during acute anabolic or catabolic challenges (e.g., with feeding or injury). To our knowledge the current work is the first to demonstrate in human beings sex differences in the rate of MPS and provides some insight concerning the control of MPS and the different rates of muscle loss with aging between men and women.

The greater basal rate of mixed MPS in older women was probably mediated by a combination of a greater capacity for protein synthesis combined with a relatively more active translational process at the elongation stage of protein synthesis because first, there was the trend for a ∼20% greater muscle RNA-to-protein ratio in women than in men, indicating a greater capacity for MPS [24] in them and secondly, muscle of older women had a 40% smaller degree of phosphorylation of eEF2^Thr56^, a molecule regulating elongation of nascent protein chains which is deactivated by phosphorylation [25]. We found no differences between sexes in the extent of phosphorylation for components of the PKB/mTOR/p70s6k signaling pathway, or the phosphorylation of elements involved in the regulation of translation initiation (eIF4E and eIF4e-BP1) at baseline (fasted). Furthermore, the expression in muscle of mRNA for myostatin and myoD, cell regulatory proteins affecting muscle size [26]–[29], was not different between the sexes, which makes it unlikely that they are involved in regulating the basal rate of MPS, although, we cannot from our data rule out differences in the muscle myostatin and myoD protein concentration (and thus in cell function). Indeed, a greater myostatin protein concentration has been found in female than male mice [40], which is consistent with the observed sexual dimorphism in muscle mass but not with our results of a lower basal rate of MPS in older men, assuming extrapolation between species is valid. Plasma glucose, insulin, and CRP concentrations were not different in men and women, so these results also provide no explanation of baseline sex differences in MPS.

The proximal, underlying, biological reasons that the sexual dimorphism in muscle protein metabolism and its control is apparently of late onset (aging, as in the present study, vs. young and middle-age adulthood [20]–[22]) are not clear but are most probably related to the changes with advancing age in the sex hormone milieu. This may at first seem counterintuitive, because men had a ten-fold excess of testosterone compared with women, with a concomitant relatively small or no difference in plasma progesterone and estradiol concentration between the sexes. Testosterone is well known to be anabolic and increases the basal rate of MPS in both healthy and hypogonadal young men [15], [16], [41]. Oddly, however, the effect of testosterone therapy in older men is unclear; it has been shown by the same group, in different studies, to either increase [42] or not to affect [43] the basal rate of MPS. This discrepancy might be a dose- or treatment duration-related phenomenon, or depend upon the extent of initial testosterone deficiency. More importantly, however, we [44] and others [45] measured the basal rate of MPS in large cohorts of healthy young (n≥22) and old (n≥22) men and found that it was not affected by old age. This suggests that the normal decline in testosterone with aging, which is small [45], probably has little effect on the basal rate of MPS. On the other hand, there is evidence from studies in rats that progesterone and estrogen inhibit MPS. Specifically, it was found that in ovariectomized rats the rate of MPS was higher than in sham-operated, intact controls and ovariectomy with either progesterone or estrogen replacement prevented the increase [17]. Thus, it appears that the basal rate of MPS probably increases after menopause due to a lack of female sex steroids, which leads to pronounced differences in the basal rate of MPS between men and women not apparent in younger adults. Moreover, these and our findings (i.e., greater basal rate of MPS in women than men despite 10-fold difference in plasma testosterone concentration) suggest that the anti-anabolic effect of female sex steroids on MPS may by far outweigh the anabolic effect of testosterone.

Another somewhat surprising finding was the fact that the postabsorptive rate of MPS in women was faster despite lower basal plasma leucine concentrations in women than in men. There is ample evidence that the rate of MPS is directly related to plasma amino acid availability [44], [46], [47], particularly that of plasma leucine [48]–[51]. However, this relationship has been established when plasma amino acid/leucine concentrations were varied several-fold, whereas the difference in plasma leucine concentration in our men and women was only ∼15%, possibly too small to exert a noticeable effect on the rate of MPS as MPS only increases by 50% with a doubling of leucine concentration [46].

The slightly greater postabsorptive plasma leucine concentration in men, which is in excellent agreement with an earlier report on sex differences in the plasma amino acid profile [52], probably reflects greater net negative protein balance in men than in women but it is difficult to assign components to this, beyond the decreased MPS in men. We did not measure the rate of muscle protein breakdown (MPB) (which was unwarranted without more indicative data of possible sex differences given the already sizeable investigational burden on our subjects). Therefore we are unable to estimate muscle protein net balance. However, whole-body protein breakdown (indicated by leucine Ra) was not different between our men and women and as MPB normally accounts for ∼20–40% of a healthy person's postabsorptive whole-body protein breakdown rate [53], [54] it is unlikely that MPB was markedly accelerated in men compared with women.

The differences in the anabolic response to feeding between the sexes, with men showing a significant increase and women no significant increase in MPS, was probably partially mediated by a lack of stimulation by feeding of protein translation initiation in female muscle, given that the feeding increased the phosphorylation of muscle eIF4E^Ser209^ and eIF4E-BP1^Thr37/46^ in men but not in women. This is particularly interesting given the facts that other indices of anabolic signaling, like the feeding-induced changes in the phosphorylation of Akt^Thr308^, p70s6k^Thr389^, and eEF2^Thr56^ were similar in men and women. An intriguing new finding was the marked (and so far as we can tell previously unreported) changes in the expression of myostatin and myoD with feeding, which are consistent with an acute nutritional control at the level of the nucleus of processes regulating muscle mass [55]. Nevertheless, there was no apparent sex difference in these responses, which makes it unlikely that the feeding-induced changes in myostatin and myoD expression contributed to the blunted increase of MPS to feeding in women. The feeding-induced increases in plasma glucose, insulin, and leucine concentrations were not different in men and women, which suggests that these also were not involved in the apparent anabolic resistance in women. It is possible, however, that there were sex differences in the insulin-stimulated increase in muscle nutritive blood flow which would differentially affect amino acid delivery to the muscle. Although in young adults, the insulin mediated vasodilation is greater in women than in men [56], there is evidence for a greater decline with aging in women compared with men in endothelium dependent dilation induced pharmacologically or by hypoxia [57]–[59], which may abolish this difference at a more advanced age or even result in reduced flow in women. However all these observations were for measures of bulk blood flow and no pertinent results exist, to our knowledge, for muscle microvascular perfusion [60]. The impact of the sex hormone milieu on the anabolic effect of feeding in muscle is unknown largely due to a lack of data in the literature. To our knowledge, no one has determined the effect of female sex steroids on the anabolic effects of amino acids or food in muscle; however, neither testosterone [43] nor oxandrolone [61] were found to exert an acute additive effect to the stimulatory effect of amino acids on MPS.

Our findings provide a potential mechanism that may help to explain the slower rate of muscle wasting during aging in women than in men [5], [7]–[9]. There is no data available for diurmal variation in human muscle MPS but we suggest that the basal, postabsorptive rate, which was ∼30% greater in our women than in the men, most likely predominates over most of the day. Our arguments depend upon a number of observations. First, the data available from protein intakes in French [62] and North American [63], [64] older adults indicate that individuals between 65–80 y eat on average approximately 9–14 g protein at breakfast, 38–64 g protein at lunch, 19–33 g protein at dinner and 1.2–1.7 g protein in snacks [62]. Secondly, meal feeding causes an increase of plasma total amino acid concentrations that is proportional to the amount of protein consumed for approximately 3–5 h, after which the change returns to within 25% of baseline [65]–[67]. Thirdly, the results of a study of the circadian variations of amino acids during a 24-h long study of the effects of 3 meals and two snacks suggested that the period of plasma essential amino acids being raised above the postabsorptive morning value was approximately 7 h [68]. Fourth, there is evidence that a sustained hyperaminoacidemia does not lead to a sustained increase in MPS, which returns to baseline values within approximately 2–2.5 h of the initial increase of amino acid availability [69]. Lastly, the relationship between amino acid availability and MPS is saturable both for orally delivered free amino acids [44] and orally delivered protein [70], with excess essential amino acids being oxidized above delivery of approximately 15 g of protein per meal in young trained individuals [70] and probably even less in older adults [44]. Thus, we suggest that MPS proceeds at a rate corresponding closely to fed values in our study for approximately 6–8 h; for the remainder of the day, MPS would presumably operate at or near postabsorptive values. This then would result in a lower than average rate of MPS over the course of the day than in women. We recognize that a complete description of the mechanisms involved in nutritional regulation of muscle mass in the elderly will depend on information about the meal induced changes in muscle protein breakdown. To date, there is no evidence that the sensitivity of MPS in older men and women to insulin released by feeding is different. Furthermore, in a study of 6 older men and 6 older women (68–70 y), who received two doses of insulin during hyperinsulinaemic, isoaminoacidemic clamps, leucine whole body Ra (an index of whole-body proteolysis) was not different between the sexes [71]. Additional support comes from a study of 30 y old men and women, in which it was observed that the inhibitory effect of insulin on whole-body proteolysis (assessed during a hyperinsulinemic, euglycemic, isoaminoacidemic clamp) was not different between the sexes but, consonant with our results, in women there appeared to be an anabolic resistance to the stimulatory effect of insulin on whole-body protein synthesis [72].

In summary, we have demonstrated that there is significant sexual dimorphism in MPS and its control in older adults; a greater basal rate of MPS, operating over a large portion of the diurnal cycle, may be, at least in part, responsible for the slower loss of muscle in women than in men.
